# Brownsburgh Medical Society

**Published:** 1859-12

**Authors:** H. H. Moore, Ross C. Russ

**Affiliations:** President; Secretary


### PROCEEDINGS OF MEDICAL SOCIETIES.

### BROWNSBURGH MEDICAL SOCIETY.

In accordance with previous notice, the physicians convened at the Odd-Fellows Hall, in the village of Brownsburgh, Ind., July 21st, 1859, for the purpose of organizing a medical society.

On motion, Dr. D. H. Oliver was called to the chair; Dr. Ross C. Russ chosen Secretary *pro tempore.*

Dr. D. H. Oliver, on taking the chair, made a brief, though quite a comprehensive, speech to the Society.

A constitution and by-laws were then presented by the committee appointed for that purpose at a previous meeting, after considerable discussion, were amended, and unanimously adopted, as the Society deemed necessary, etc.

An election for permanent officers being the next thing in order, resulted in the election of the following named gentlemen, for one year, viz: Dr. H. H. Moore, President; Dr. William J. Hoadley, Vice-President; Dr. Ross C. Russ, Secretary. Drs. A. J. Graham, T. P. Seller and M. Clark Russ, Censors.

Drs. J. P. Graham and D. H. Oliver were appointed by the President essayists for next meeting.

On motion of Dr. D. H. Oliver, that the Secretary furnish the *Cincinnati Lancet and Observer* and *Chicago Medical Journal*, each a copy of the proceedings of this meeting, that they might be published at the request of the Society.

On motion, the Society then adjourned, to meet in Brownsburgh on the first Thursday in January next, at 10 o'clock A.M.

H. H. Moore, *President.*

Ross C. Russ, *Secretary.*

